# Integrated Metabolomic and Transcriptomic Analysis of Volatile Organic Compound Biosynthesis During Mung Bean (*Vigna radiata*) Seed Development

**DOI:** 10.3390/foods14132183

**Published:** 2025-06-22

**Authors:** Nan Xiang, Yihan Zhao, Bing Zhang, Honglin Chen, Xinbo Guo

**Affiliations:** 1 College of Biology and Food Engineering, Guangdong University of Education, Guangzhou 510303, China; nanxiang0908@163.com; 2 School of Food Science and Engineering, South China University of Technology, Guangzhou 510641, China; zhaoyihan19980515@163.com (Y.Z.); dbkxydd@163.com (B.Z.); 3 Institute of Crop Sciences, Chinese Academy of Agricultural Sciences, Beijing 100081, China

**Keywords:** mung bean, seed development, volatile, transcriptomic, transcription factor

## Abstract

Mung bean (*Vigna radiata* L.) is globally cultivated and has been widely used in the food industries. Other than nutrients, the composition of the volatile organic compounds (VOCs) often influences the quality of mung bean-based products. However, the dynamics of VOCs and the flavor changes during mung bean seed development remain unexplored. This study investigated the VOC and flavor composition in four mung bean varieties by integrating relative odor activity value (ROAV) evaluation and transcriptomic analysis. A total of 65 VOCs were identified, with eucalyptol serving as a key maturity indicator in LL655 and SH-1, while nonanal contributed significantly to the characteristic beany flavor across all varieties. Transcriptomic analysis revealed four downregulated geranylgeranyl diphosphate synthase genes during seed development, leading to terpenoid accumulation patterns. Terpenoids, including trans-beta ocimene and gamma-terpinene, appeared to be regulated by transcription factors (TFs) from the RLK-Pelle, WRKY, AP2/ERF, bHLH, and bZIP families. Additionally, two MYB TFs showed potential roles in modulating the accumulation of phenylpropanoid/benzenoid derivatives. This study provides a comprehensive understanding of the VOC accumulation and flavor variation during mung bean seed development, enriches the knowledge of flavor chemistry in mung bean varieties, and facilitates a theoretical foundation for optimizing and developing mung bean-based products.

## 1. Introduction

Mung bean (*Vigna radiata* L.), a globally cultivated legume with over 3500 years of consumption history, currently occupies 8.5% of the world’s legume cultivation area, which is more than 6 million hectares, with a short maturation cycle of approximately 70 days [1,2]. Its expanding industrial applications from nutritional and pharmacological properties meet the needs of chronic disease prevention and nutritious dietary supplementation [1,2]. In food industries, in particular, mung bean serves as raw material for vegetable protein beverages and milk, fermented dairy analogues, and starch-based products [3]. Beyond macronutrients, volatile organic compounds (VOCs) play decisive roles in the end-product quality by critically determining the flavor formation and odor characteristics during food processing procedures [4].

The profiling of VOCs holds significant economic and sensory importance, enabling the identification of off-odors and quality grading of mung beans [5]. The main VOCs in mung bean are alkenes, esters, and alcohols, while the traditional beany flavor primarily comes from hexanal, (E)-2-hexenal, hexanol, 1-octe ne-3-ol, and (E,E)-2,4-decadienal [3,6]. Current research categorizes VOCs into three compound categories: terpenoids, phenylpropanoid/benzenoid derivatives, and fatty acid derivatives. As reported, some alcohols, aldehydes, acids, esters, and ketones are oxidized from fatty acids [7]; hence, the beany flavor VOCs mainly originate from fatty acid degradation, yet the sources of others still need to be investigated. Therefore, clarifying the formation mechanisms of key VOCs in mung bean is conducive to improving the overall quality and stability of mung bean-based food products.

Having such a short growing period, the promising predicted yield of mung bean ranges from 2.5 to 3.0 t/ha, however, mung bean exhibit susceptibility to various insects and diseases during seed development, which leads to its surprisingly low yield at 5000 kg/ha [8]. Notably, terpene VOCs are pivotal in plant defense by mitigating plant damage symptoms, defending against pests, and attracting the natural enemies of insects, such as (*E*)-nerolidol in tea plants [7]. Furthermore, VOCs, such as terpenoids and phenol-derived aromatic components, have attracted significant attention from their applications in various industries, including serving as food additives for imparting specific aromas, materials for industrial pharmaceuticals, and components for biofuels [9]. Therefore, systematic investigation of VOC accumulation kinetics during mung bean seed development can not only contribute to elucidating the biosynthetic pathway, but it can also lay the foundation for yield optimization, as well as volatile-based ingredients exploration.

To date, the metabolic dynamics of VOCs during mung bean seed development remain underexplored. While previous studies have characterized anthocyanin biosynthesis in black mung bean seed coats [10] and the starch properties of mung bean seeds during development [11], critical gaps persist in understanding how VOCs and flavor change or accumulate, limiting the manipulation of flavor profiles during mung bean seed development. Therefore, by combining transcriptomic analysis with the VOC profiles across four popular consumed mung bean varieties through three developmental stages, this study identified important flavor compounds through relative odor activity values (ROAVs), characterized the VOC metabolic pathway, as well as investigated potential transcription factors. This work provides broadened knowledge governing VOCs, flavor regulation and accumulation, and establishes a foundation for improving mung bean quality and facilitates novel strategies for developing volatile-driven ingredients.

## 2. Materials and Methods

### 2.1. Sample Collection

Four mung bean varieties (ZL-5, ZL-13, LL655, and SH-1) were planted in artificial climate chambers with uniform settings (25 °C, 80%, and 12 h/12 h). Seeds were collected at three developmental stages: beginning seed (BS), full seed (FS), and beginning maturity (BM) (Figure S1). At the BS stage, around 6–7 days after anthesis, the seeds were initially formed, with a diameter of about 2 mm, showing a soft green translucent shape and an inconspicuous seed coat; at the FS stage, around 9–11 days after anthesis, the seeds bulged to their maximum size as 8–10 mm in diameter with a discernible seed coat, and the pods showed a green color; at the BM stage, around 13–15 days after anthesis, the seeds reached maturity, and the seed coats showed characteristic color according to the different varieties. Three biological replicates per stage were set up for each mung bean variety. After collecting, the mung bean seeds were immediately frozen in liquid nitrogen and stored at −80 °C for subsequent experiments.

### 2.2. Transcriptomic Analyses

The total RNA was extracted using an RNA Plant Tissue Kit (Tiangen Biotech Co., Ltd., Beijing, China). After quality verification, the mRNA was processed for library preparation using poly (A) selection with oligo (dT) beads, followed by mRNA fragmentation, cDNA synthesis with random hexamer primers, end repair, A-tailing, and adapter ligation. The final libraries were quantified by PCR (effective concentration > 2 nM). Pooled libraries were sequenced on the Illumina platform in paired-end 150 bp mode, generating approximately 5.73 Gb clean data, and the percentage of Q30 bases reached 92.73%. Sequence comparison efficiency ranged from 91.39–96.50%. Predictive analysis of variable splicing, optimization analysis of gene structure, and the discovery of new genes were all carried out based on the comparison results, and a total of 3536 new genes were discovered, of which 1898 new genes were functionally annotated. Differential expression analysis used DESeq2 with the following thresholds: |log2FC| ≥ 1 and FDR < 0.01.

### 2.3. Volatile Organic Compounds (VOCs) Determination

VOC determination was operated by the GC-MS/MS method. Sample was added in a headspace bottle and went through the heat processing of a gas chromatography system (HS-SPME-GC, 7890B, Aglient Technologies Inc., Santa Clara, CA, USA). VOCs were absorbed by a 15 μm PDMS/DVB fiber (Anpel Laboratory Technologies Inc., Shanghai, China) and were then separated in a DB-Wax column (30 m × 250 μm × 0.25 μm, J&W Scientific Inc., Folsom, CA, USA) using helium at a 1 mL/min flow rate. The programs were set with modification according to a previous method [12]. A triple quadrupole-MS (TQ-MS, 7000C GC/MS Triple Quad, Aglient Technologies Inc., Santa Clara, CA, USA) was subsequently equipped for compound analysis. The electron-impact mass spectra was 70 eV with a scan range from 33 to 475 *m*/*z*. The emission current was 100 μA, and the detection voltage was 1.4 kV. L-2-octanol (Shanghaiyuanye Bio-Technology Co., Ltd., Shanghai, China) was used as the internal standard for the semi-qualification of the VOCs. Results are expressed as the ng·g^−1^ fresh weight (FW) in triplicate.

### 2.4. Weighted Gene Co-Expression Network Analysis (WGCNA)

WGCNA was conducted on the BMKCloud platform (www.biocloud.net). Genes with FPKM ≥ 1 were screened from 36 samples. The minimum module size was 30, and the minimum height of the merged modules was 0.25, both of which guarantee the high reliability of results. The correlation analysis was conducted according to the biweight midcorrelation or the Spearman correlation [13].

### 2.5. Flavor Evaluation

In order to assess the change in the main flavor in the mung bean seeds, the odor activity value (OAV) and the relative odor activity value (ROAV) of each volatile compound that had an odor threshold [14] were calculated according to the following formulas:

OAV = C/T,

ROAV = C/C_max_ × T_max_/T × 100%,

where C and T represent the content of each VOC and the threshold value of each VOC, respectively; and C_max_ and T_max_ represent the relative amount and threshold of the component that contributes the most to the total odor of the sample, respectively.

### 2.6. Statistical Analysis

Data were primarily calculated and plotted using OriginPro 2023 (Origin Lab Corporation, Northampton, MA, USA). Correlation analysis, cluster analysis, and significance analysis (*p* value < 0.05) were performed using IBM SPSS 25.0 (SPSS Inc., Chicago, IL, USA). Transcriptomic analysis was performed on BMKCloud (www.biocloud.net), including KEGG enrichment and transcription factor prediction. Venn diagram analysis was performed on jvenn [15]. Three replicates were done for all determinations.

## 3. Results

### 3.1. Transcriptomic Profiling Revealed Stage-Specific Gene Expression Patterns

As shown in Figure S2A, principal component analysis (PCA) of the transcriptomic data separated samples into different quadrants, exhibiting the large disparity of gene information in the different developmental stages of mung bean seeds. The gene expression profiles of different mung bean varieties demonstrated greater similarity when compared within the same developmental stage, which indicates that the genetic variations among these varieties were relatively limited. However, when comparing samples across different developmental stages, separation in gene expression patterns was observed, revealing that the transcription changed remarkably throughout seed development. Figure S2B exhibits the correlation value among the samples, supporting the high parallelism of each studied group (*r* > 0.94).

Quantitative analysis of the expressed gene in Figure S3A showed a gradual decrease in the total gene number from the BS to BM stages. In detail (Figure S3B), high-abundance transcripts (FPKM ≥ 10) predominated at BS in LL655, SH-1, ZL-5, and ZL-13 as 25.61%, 27.03%, 25.65%, and 29.19%, respectively, but they declined to 18.16%, 17.06%, 17.65%, and 17.06% by BM, respectively. Conversely, low-abundance genes (FPKM ≤ 1) increased at the BM stage by 14.21%, 11.74%, 13.84%, and 13.50%, respectively. The quantity of the expressed genes in the four mung bean varieties during seed development were further characterized by a Venn plot (Figure S3C). In total, 11,201 genes were identified in all four varieties, with SH-1 exhibiting the highest varietal specificity (991 unique genes) versus ZL-13 (167 unique genes), which may be related to the seed coat color changes in the late stage of the seed. The number of genes expressed in all three stages was 13,436, 14,237, 13,183, and 12,875 for LL655, SH-1, ZL-5, and ZL-13, respectively (Figure S3D). The number of specially expressed genes in the four mung bean varieties decreased and then increased during seed development, and the highest number was in ZL-13 as 3923 at the BS stage.

### 3.2. Differential Gene Expression Dynamics

**Figure 1:**
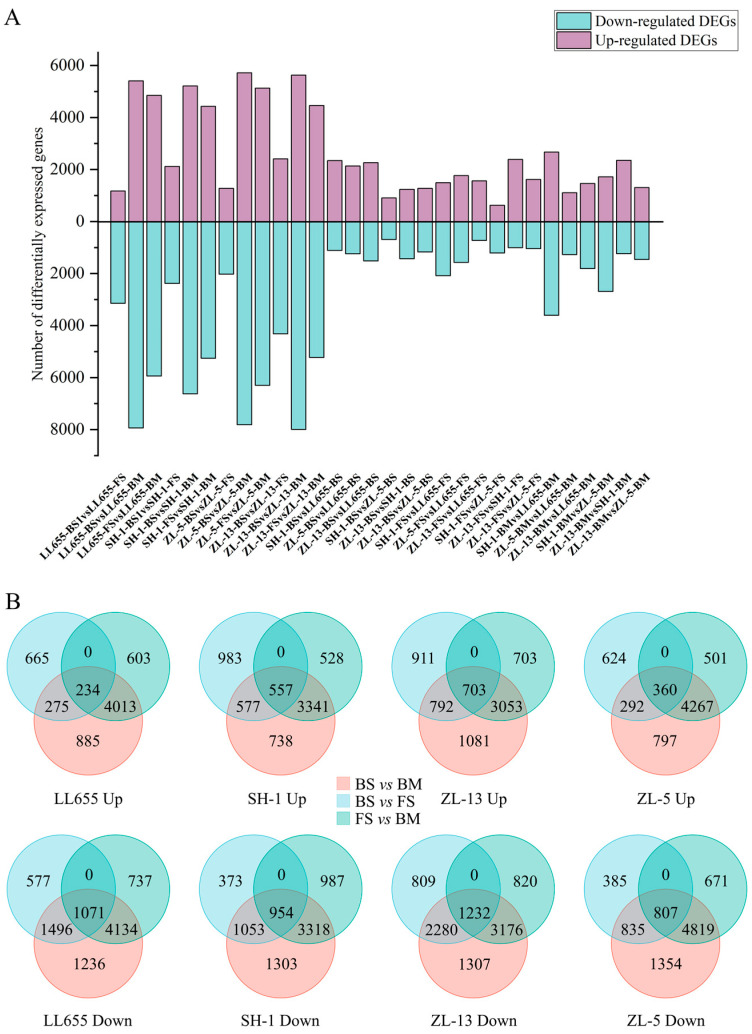
The analysis of differentially expressed genes (DEGs). (**A**) Number of DEGs in each compared group; (**B**) Venn plot of the DEGs in each mung bean variety. BS: beginning seeds; FS: full seeds; and BM: beginning maturity.

Pairwise comparison of the developmental stages was analyzed to create differentially expressed genes (DEGs) using the BS and FS stages as controls, respectively, and the results were represented in bar charts (Figure 1A) and Venn plots (Figure 1B) (|log2FC| ≥ 1, FDR < 0.01). From the BS to FS stages, the numbers of downregulated genes in the four varieties of mung bean were higher than that of the upregulated genes, indicating the decreased expression levels of most of the genes. With the processing of seed development, the numbers of both the up and downregulated DEGs in the four varieties of mung bean unanimously increased. The numbers folded to more than 4000 in the comparison of the BM and FS stages, expressing an activation of the gene expression in the mung bean seeds. A similar situation was observed during peanut pod development [16]. In addition, seed development changed the gene differences among the varieties. When comparing LL655 to SH-1, an increasing number of DEGs were found in the downregulated category during seed development, while the number was slightly decreased and then increased in the upregulated category, which was probably related to the different seed appearance at the BM stage. The same phenomenon was also detected when comparing ZL-5 to SH-1. The number of upregulated DEGs kept decreasing in ZL-5 vs. LL655, as well as in ZL-13 vs. LL655. These results indicate the variety difference at the transcriptional level, providing basic information for relative analysis. Figure 1B separately depicts the Venn plot of each variety. In LL655, 234 and 1071 DEGs were consistently up or downregulated during seed development, respectively. The numbers changed to 557 and 954; 703 and 1232; and 360 and 807 in SH-1, ZL-13, and ZL-5, respectively, while ZL-13 reached the highest with 1935 DEGs in total. The above results indicate that all the four varieties of mung bean seeds are metabolically active during the developmental period, with a greater number of highly expressed genes and specifically expressed genes.

The obtained DEGs were analyzed by KEGG functional annotation, and the results are shown in Figure S4. Typically, the downregulation of genes dominated the metabolism category during seed development, while upregulation prevailed in the pathways categorized in genetic information processing. In the metabolism category, starch and sucrose metabolism, carbon metabolism, and biosynthesis of amino acids were the most active processes during mung bean seed development. In addition, the KEGG annotation includes metabolic pathways that are related to volatile generation and degradation, such as phenylpropanoid biosynthesis.

### 3.3. Identification of Transcription Factors That Regulate Seed Development

**Figure 2:**
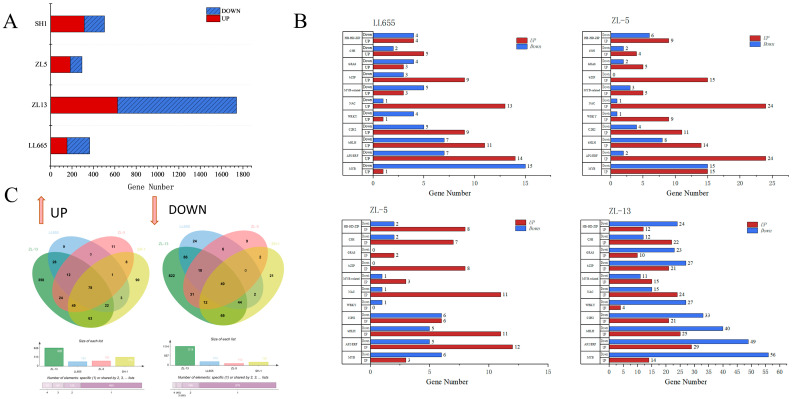
The results of the TF annotation in the DEGs during seed development. (**A**) The annotated TF number in each mung bean variety; (**B**) the annotated TF number according to the TF families in each mung bean variety; and (**C**) the Venn plot of the TF in the up and downregulated categories among the four mung bean varieties.

Transcription factors (TFs) serve as a crucial bridge for transmitting upstream signals to downstream genes and for precisely regulating their expression. There are multiple TFs in plants that regulate biological processes by controlling the expression of downstream target genes and participating in the entire life cycle of plants. In addition, TFs are also key regulatory factors that control the gene expression in plants’ response to biotic and abiotic stress [17]. The above-selected DEGs were classified and statistically analyzed according to TF families. A total of 4013 differentially expressed TFs were annotated to 68 families, with MYB, AP2/ERF, bHLH, C2H2, RLKs, WRKY, NAC, and bZIP being the most abundant. As shown in Figure 2A, ZL-13 had the highest number of TFs. More than half of the TFs were downregulated in LL655 and ZL-13, while more were upregulated in ZL-5 and SH-1. Classification was conducted on several major TF families (Figure 2B). Among the major TF families, AP2/ERF had the highest number of upregulated DEGs, while MYB showed the highest number of downregulated DEGs, which is consistent with the TF changes during the development of young pomelo [18]. According to the Venn plot (Figure 2C), 78 consistently upregulated and 40 consistently downregulated TFs, which may participate in the core seed development process, were identified across all varieties, while the varietal-specific TFs may have contributed to the unique seed properties.

### 3.4. Dynamic Changes in Volatile Organic Compounds (VOCs)

There is a lack of investigation in how mung bean seeds accumulate VOCs during seed development; hence, the present work studied the VOC profiles during mung bean seed development of the chosen four mung bean varieties using GC-MS/MS. As shown in Figure S5, 65 VOCs were detected across four mung bean varieties, including acids, amines, aromatic hydrocarbons, alkenes, alkanes, alcohols, ketones, aldehydes, esters, and terpenoids. The VOC composition varied significantly among varieties, with LL655 having the most compounds at 45, followed by 35 in ZL-13, 18 in ZL-5, and 17 in SH-1. The dominant VOCs changed during development. Eucalyptol was most abundant in LL655 at the BS stage, while toluene dominated at later stages. Similarly, toluene showed the highest levels throughout development in SH-1. In ZL-13 and ZL-5, cis-2-penten-1-ol and toluene were the major compounds at the BS stage, respectively, but their relative abundance shifted in later stages. In general, the terpenoids represented a significant portion of the VOCs in the mung bean seeds, with 14 compounds identified.

**Figure 3:**
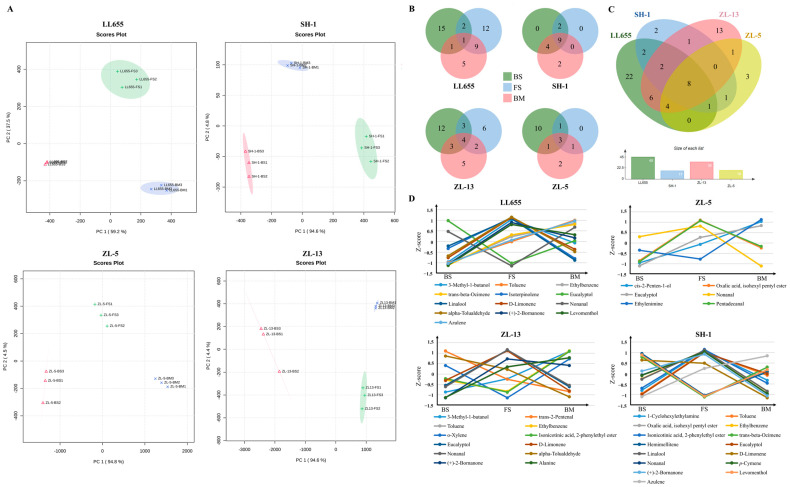
The volatile organic compound (VOC) profiles in the detected mung bean seeds. (**A**) PCA analysis of the VOCs in each mung bean variety; (**B**) Venn plot of the VOCs according to the developmental stage in each mung bean variety; (**C**) Venn plot of the detected VOC compositions; and (**D**) the accumulating pattern of the *z*-score standardization of the VOC content during seed development in each mung bean variety. BS: beginning seeds; FS: full seeds; and BM: beginning maturity.

PCA revealed a clear separation of the samples by developmental stage (Figure 3A), while Venn analysis demonstrated the dynamic changes in the VOC numbers across stages (Figure 3B). LL655 showed an initial increase from 19 VOCs at the BS stage to 24 at the FS stage, followed by a decrease to 16 at the BM stage (*p* < 0.05). Only nonanal was stably preserved during the developmental process. Different from LL655, SH-1 maintained relatively stable VOC numbers with 15 at the BS and BM stages and 11 at the FS stage, with nine compounds consistently present across all stages, including nonanal, some phenylpropanoid derivatives, and monoterpenoids. In ZL-13, the number of VOCs exhibited a progressive decrease from 22 to 14, while 4 VOCs were retained during the development period. The number of VOCs was the highest at the BS stage of ZL-5 at 15; with the process of seed development, however, the number sharply decreased to 5 at the FS stage and then slightly increased to 7 at the BM stage. Only cis-2-penten-1-ol, oxalic acid, isohexyl pentyl ester, and eucalyptol could be detected throughout the entire period. Among all the varieties, 8 conserved VOCs were identified, including the phenylpropanoids/benzenoid derivatives ethylbenzene and toluene; the fatty acid esters oxalic acid, isohexyl pentyl ester, and isonicotinic acid; 2-phenylethyl ester; aldehyde nonanal; and the monoterpenoids trans-beta-ocimene, eucalyptol, and D-limonene (Figure 3C).

The VOCs that could be detected in more than one stage are screened from the results and depicted in Figure 3D according to developmental stages. Contents went through a z-score standardization. Specifically, in LL655, the levels of the phenylpropanoids/benzenoids like toluene and ethylbenzene, monoterpenoid trans-beta-ocimene, and naphthalene azulene, showed continuous increases during seed development. Eucalyptol and nonanal separately dropped to their lowest content in the FS stage, while other screened volatiles achieved the highest level. Similar to LL655, SH-1 exhibited increasing azulene but decreasing D-limonene levels with developing progress. Different from LL655, toluene and trans-beta-ocimene showed different variation trends in SH-1, reaching the smallest value in the FS stage. Alcohol 3-methyl-1-butanol and amino acid alanine accumulated in ZL-13 during seed development; meanwhile, the value of aldehydes trans-2-pentenal and phenylpropanoids/benzenoid derivative alpha-tolualdehyde dropped to the lowest in the BM stage. In ZL-5, the increasing trends were only detected in cis-2-penten-1-ol and eucalyptol as the content of the other three fatty acid derivatives increased from the BS to FS period but dropped to lower levels in the BM stage.

### 3.5. Modulation of the VOC Accumulation During Mung Bean Seed Development

The accumulation patterns of the VOCs during seed development in the four varieties were influenced by multiple factors, including genetic variation and metabolic regulation. To understand the molecular mechanisms underlying VOC biosynthesis, WGCNA was performed on the VOCs and genes from the transcriptomic results. Generally, the genes were classified into two modules: MEblue and MEturquoise (Figure S6A). Thirteen VOCs showed a high correlation value with the modules as *r* > 0.90 in total (Figure S6B). Functional annotation revealed distinct metabolic pathways associated with each module. Notably, phenylpropanoid biosynthesis showed the highest number of associated genes in both modules (MEblue: 127 genes; MEturquoise: 14 genes), with 65 genes consistently downregulated across all varieties (Figure S6D).

KEGG functional annotation was operated on the genes from the two modules (Figure S6C). As has been shown, 11, 16, and 11 genes in the MEblue module were classified into alanine relative metabolism, phenylpropanoid biosynthesis, and fatty acid degradation, respectively. Furthermore, several of the annotated genes that belong to pathways like diterpenoid biosynthesis and alpha-linolenic acid metabolism were found to be related to VOCs. In the MEturquoise module, 42 and 125 genes were annotated in phenylalanine and phenylpropanoid biosynthesis. Figure S6D classifies the VOC relative genes into the up and downregulation categories, as well as those mainly involved in the biosynthesis of fatty acids, phenylpropanoid biosynthesis, and terpenoid backbone biosynthesis. Notably, phenylpropanoid biosynthesis showed the highest number of associated genes in both the MEblue and MEturquoise modules at 127 and 14, respectively. The majority of the genes were downregulated in the MEturquois module. In the phenylpropanoid biosynthesis pathway, 65 genes were downregulated, accounting for more than half of the total downregulated genes, while it was at its lowest in the fatty acid biosynthesis pathway at 15. Diversely, most genes showed upregulation patterns during seed development in the MEblue module.

**Figure 4:**
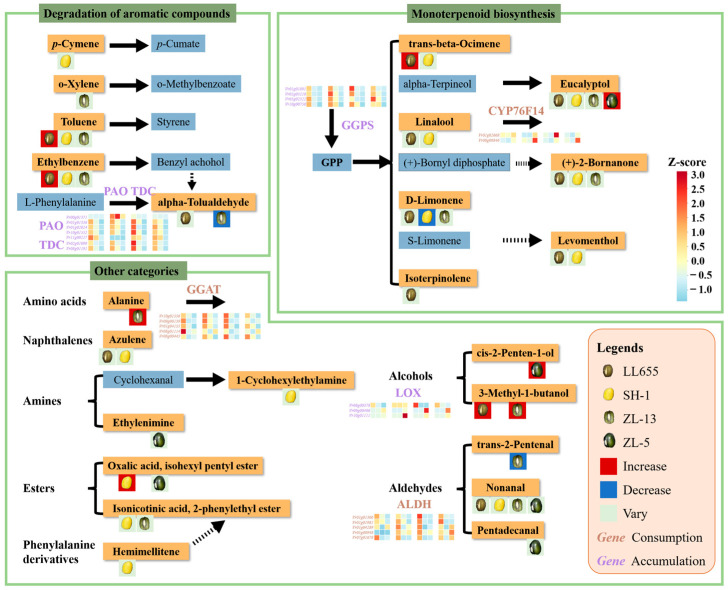
The predicted VOC biosynthesis pathway and relative DEGs. The VOCs detected in the present study are depicted in orange frame, while the others are in blue frame. The genes involved in VOC accumulation are shown in purple, while the consumed relative genes are marked in brown. Red, blue, and green backgrounds of the mung bean seeds patterns separately represent the VOC variation during the seed development in each mung bean variety.

Further analysis identified key structural genes controlling specific VOC biosynthesis pathways (Figure 4). For the phenylpropanoid derivatives, seven genes separately encoding L-tryptophan decarboxylase (TDC) and primary-amine oxidase (PAO) showed coordinated downregulation in ZL-13, corresponding to decreased alpha-tolualdehyde levels. Monoterpenoids are terpene derivatives from the transformation of geranylgeranyl diphosphate (GGPP), which synthesizes from methylerythritol phosphate and mevalonate pathways via the catalyzation of geranylgeranyl diphosphate synthase (GGPS). Four GGPS encoding genes were detected with progressive downregulation during development in the four mung bean varieties. This pattern explains the observed accumulation of the monoterpenoids from the BS to FS stages, which was followed by a decline at the BM stage. The high expressional levels of *GGPSs* accumulated GGPP at the BS stage, which was then consumed via spontaneous reactions to generate monoterpenoids, leading to increases in monoterpenoid content at the FS stage. Subsequently, with the downregulation of *GGPSs* during seed development, a low level of GGPP resulted in a decrease in the monoterpenoid content at the BM stage. In both L655 and SH-1, there was upregulated *CYP76F14* encoding (*E*)-8-carboxylinalool synthase that catalyzed linalool; hence, the linalool content showed an increase trend from the BS to FS stages, and it then dropped in the FS to BM stages. The dynamic expression of *CYP76F14* and *GGPS* reasonably modulated the variation in the linalool in this two mung bean varieties. Generally, the glutamate-glyoxylate aminotransferase (GGAT)-encoded genes were downregulated during seed development and controlled the consumption of alanine. The increased alanine level in ZL-13 was in line with the reduced expressional values of *GGATs*. Lipoxygenase (LOX) and aldehyde dehydrogenase (ALDH) are essential for the generation of fatty acid derivative volatiles. In ZL-5, the upregulated *Vr09g00486* and *Vr10g01111* genes corresponded with increasing cis-2-penten-1-ol levels, while similar expression patterns in ZL-13 were associated with 3-methyl-1-butanol accumulation. These findings provide new insights into the genetic regulation of VOC biosynthesis during mung bean seed development.

### 3.6. Transcriptional Regulation of Volatile Accumulation

**Figure 5:**
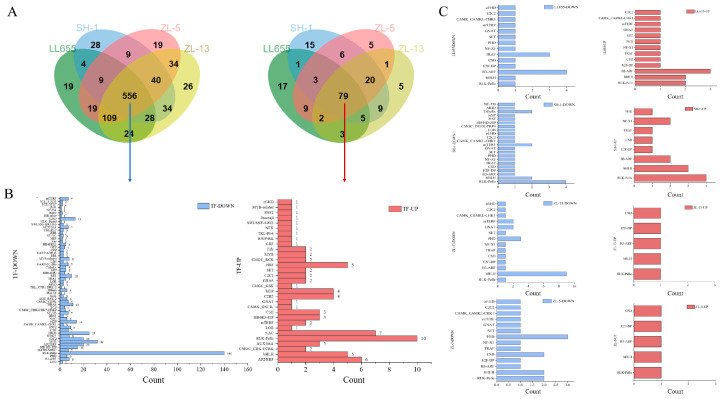
The results of the TF annotation in the MEturquoise module. (**A**) Venn plot of the TF in the up and downregulated categories; (**B**) the TF number according to the TF families in the up and downregulated categories; and (**C**) the number of TFs that were uniquely detected in the different mung bean varieties.

The prediction of transcription factors (TF) was operated and identified 871 TFs belonging to the MEturquoise module, which were classified into upregulated and downregulated categories (Figure 5A, Table S1). In the Venn plot, most of the TFs showed consistent expression patterns across all four varieties, with 556 downregulated and 79 upregulated. The RLK-Pelle family, known for its important role in plant development, contained the highest number of both upregulated and downregulated TFs (Figure 5B). Varietal-specific TFs are also identified in Figure 5C. LL655 showed 19 downregulated and 17 upregulated TFs, predominantly from the B3-ARF family. SH-1 contained 28 uniquely downregulated and 15 upregulated TFs, with RLK-Pelle being the most abundant family in both groups. ZL-13 had 28 uniquely downregulated TFs, mainly from the bHLH family, which is involved in plant secondary metabolism, including terpenoid biosynthesis. Similar to ZL-13, a small number of TFs were annotated in the upregulation group in ZL-5. ZL-5 showed 19 uniquely downregulated TFs, with the PHD family being most abundant. These varietal-specific TFs likely contributed to the distinct VOC accumulation patterns observed in each mung bean variety. Although the biosynthesis and degradation pathways of many VOCs remain poorly understood, the identified TFs may play key regulatory roles in the accumulation of specific compounds, such as ethylbenzene, trans-beta-ocimene, and *p*-cymene, which are associated with the MEturquoise module.

## 4. Discussion

### 4.1. Flavor Variation Among the Mung Bean Varieties

According to previous studies, the beany flavor in the legume that has been primarily attributed to aldehydes, such as (E)-2-hexenal, hexanal, nonanal, and (E)-2-nonenal [19], plays a crucial role in consumer acceptance. However, excessive levels of these VOCs can negatively impact product quality. Therefore, understanding the formation and controllable point of VOCs during seed development is conducive to the production of satisfactory mung bean products. In this study, we observed distinct VOC accumulation patterns across four mung bean varieties, with 27 of the 65 detected volatiles having known odor threshold values in water [14], which were calculated for the odor activity value (OAV) and relative odor activity value (ROAV) of the flavor compounds, as shown in Table 1. Apart from the compounds making the greatest contribution (ROAV = 100) to the odor of mung bean seeds, the VOCs with a ROAV larger than 1 were also regarded as significant contributors to the odor. VOCs with a ROAV ranging from 0.1 to 1 are regarded as having modifying effects on the flavor, while <0.1 compounds only have potential aroma contributes [20]. In addition, the odor description of compounds was recorded, as shown in Table S2, according to three online references: PubChem (pubchem.ncbi.nlm.nih.gov), FEME (www.femaflavor.org), and Preflavory (www.perflavory.com).

LL655 exhibited the most complex flavor profile. At the initial BS stage, four VOCs served as main contributors (ROAV value > 1), with trans-beta-ionone (woody aroma) dominating. Over the process of seed development, the number of contributors increased to seven at the FS stage, while linalool ranked the first, providing floral and citrus odor. Then, the ROAV values of five VOCs surpassed one, and the eucalyptol primarily presented a camphor-like and herbal odor at the BM stage. Consequently, the FS stage showed the richest aroma contributors, suggesting good flavor sources. Notably, trans-beta-ionone, which is involved in the carotenoid degradation pathway, has long been considered a fragrance ingredient and is used in the food and cosmetic industries [21]. Coincidentally with its decreased pattern from the BS to FS stages, the DEGs related to its biosynthesis were unanimously downregulated. Linalool is abundant in cocoa beans with citrus-like and floral odors [22], arising its specific role as an extremely important perfume material [23]. Its biosynthesis and transcriptional regulation were reviewed by Zhang et al. [23] via the regulation of the TFs from the MYB and ERF families on *TPS* genes. Conversely, the present results show that the linalool content was modulated by *GGPS* and its downstream gene *CYP76F14*, enlarging the regulation on linalool biosynthesis in the mung bean seeds. In addition, nonanal, with its beany flavor, occupied an increasing part during seed development, bringing a pleasant beany quality to LL655.

Conversely, ZL-5 and ZL-13 were found to be consistently dominated by eucalyptol during seed development. The odorous VOCs were less in ZL-5, where only 3, 2, and 2 VOCs held a ROAV of more than 1 from BS to BM stage. From the BS to FS stage, nonanal in ZL-5 held a ROAV of over 1, however, a burnt compound 3-methyl-1-butanol served as a main contributor instead of nonanal at the BM stage, leading to an inferior flavor quality in this variety. In ZL-13, although eucalyptol occupied the largest proportion of seed flavor during the developmental period, another seven VOCs that possessed a ROAV of >1 presented a complicate flavor composition at the BS stage. However, the number dropped to three in the later stages. Conversely, in the BS and FS stages of SH-1, linalool offered the largest proportion of seed flavor, but eucalyptol turned to be the decisive compound at maturity. Notably, nonanal served as a main contributor across all varieties. These aldehydes were identified as odor-active compounds [24], providing a pleasant beany aroma in mung bean seeds.

**Figure 6:**
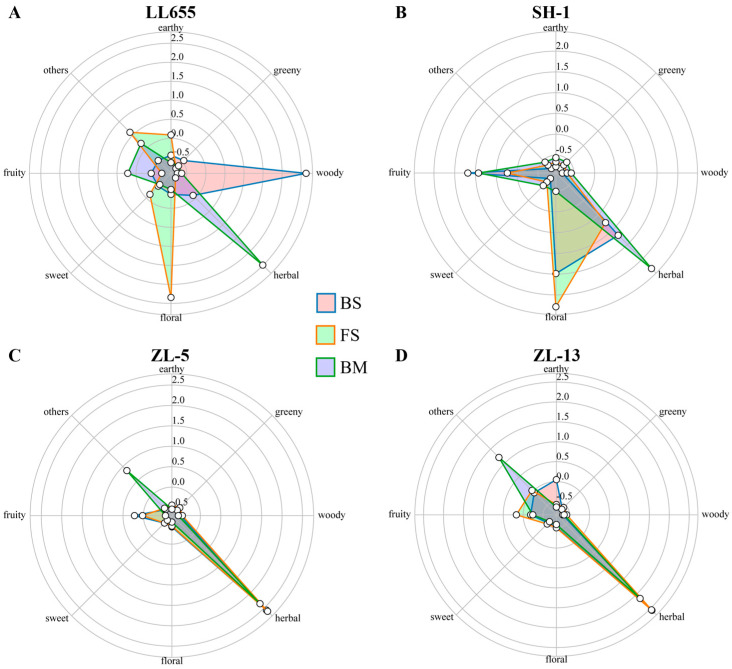
Radar plot of the odor variation during the seed development of each mung bean variety. (**A**) Radar plot of LL655; (**B**) Radar plot of SH-1; (**C**) Radar plot of ZL-5; (**D**) Radar plot of ZL-13; BS: beginning seeds; FS: full seeds; and BM: beginning maturity.

Aiming at understanding the exact flavor and quality of mung bean seeds during their development, the OAV value was used and standardized to depict a radar plot, exhibiting the main present flavor of the four mung bean varieties at each stage (Figure 6). During seed development, LL655 continuously presented woody, floral, and herbal flavor. Similarly, in the BS and FS stages, SH-1 was depicted with floral and herbal flavor, which was substituted by a complete herbal scent. ZL-5 and ZL-13 showed a similar dynamic flavor trend at each developmental period. In general, an herbal flavor, which mainly consists of a camphor-like odor, could be set as a sensory threshold to represent the maturity of LL655 and SH-1. The different odors of the LL655 and SH-1 varieties in the BS and FS stages provide extensive opportunities for processing them as raw materials to develop aromatic products. The current findings are limited by literature-based odor thresholds and the three developmental stages, suggesting future studies with cultivar-specific sensory evaluation and more frequent sampling could better characterize the VOC dynamics during seed maturation.

### 4.2. The Potential Regulatory Role of TFs on Volatile Formation

Over the last few years, a large number of studies have enhanced the understanding of plant VOC biosynthesis, transferring the emphasis from the genes encoding candidate enzymes to transcriptional regulation, epigenetics, and polyploidy [25]. In the transcriptional regulation category, a growing number of TFs have been reported as being involved in the regulation of VOC biosynthesis, including members from the bHLH, bZIP, AP2/ERF, NAC, MYB, and WRKY families [26]. Previously, the RLK-Pelle_CrRLK-1, RLK-Pelle_LRR-l-1, and RLK-Pelle_DLSV families have been reported as containing the most abundant TFs related to terpenoid biosynthesis in *Artemisia argyi*, indicating their important roles in plant growth and development [27]. In the present results, the RLK-Pelle TF family was annotated with the largest number of TFs, which might be associated with the terpenoid biosynthesis in mung bean seeds. In addition, by analyzing the co-expression network, Qin et al. [28] indicated the regulatory role of 26 WRKY TFs on 34 *TPSs* in sacred lotus. Furthermore, the annotated AP2/ERF, WRKY, bHLH, and bZIP families were relative to terpenoid biosynthesis [29]. In *Lilium*, *LiMYB305* could bind to the encoded gene of ocimene synthase, indicating its regulatory role in monoterpene biosynthesis [30]. Typically, the correlation results, as presented in Figure S6B, showed that the MEturquoise module was correlated with trans-beta-ocimene and gamma-terpinene. Trans-beta-ocimene is generated from GPP under the catalyzation of GGPS; hence, the TFs annotated in LL655 and SH-1 might affect the expression of its encoding genes during mung bean seed development. In addition, the unique TFs in LL655 and SH-1 have a potential regulatory role on different dynamic trans-beta-ocimene levels, contributing to the formation of the floral aroma in mung bean seeds.

The biosynthesis of the phenylpropanoid/benzenoid derivatives is related to their precursor phenylalanine, which is produced via the shikimate pathway and arogenate pathway [26]. Members from the MYB family have been widely studied and documented with the ability to regulate the shikimate pathway [31]. In the present study, many phenylpropanoid/benzenoid derivatives were detected in the mung bean seeds, such as o-xylene, toluene, and ethylbenzene. In particular, ethylbenzene showed a high correlation with the MEturquoise module, as shown in the results of Figure S6B, and it kept accumulating in LL655 during development, whereas a TF from the MYB family was upregulated (GenID: *Vr05g00708*), and it is named MYB3R-1-like. Another TF, annotated as MYB2-like (GenID: *Vr03g01704*), showed a high correlation with both the ethylbenzene and *p*-cymene variation in SH-1 (*r* = 0.71 and 0.80, respectively, Table S3). Therefore, those TFs may be related to the biosynthesis of the phenylpropanoid/benzenoid derivatives in mung bean seeds. While these correlations suggest potential regulatory roles, further functional validation will be necessary to confirm the direct involvement of these TFs in volatile biosynthesis.

## 5. Conclusions

In conclusion, this study comprehensively investigated the formation of volatile organic compounds (VOCs) in four varieties of mung bean seeds during their developmental stages via integrated transcriptomic and ROAV evaluation. Among the studied varieties, LL655 had the most abundant VOCs at 45, including 11 terpenoids. Eucalyptol, characterized by its camphor-like odor, served as a key maturity indicator in both LL655 and SH-1, while these varieties also showed potential as valuable sources of fragrance ingredients, such as linalool and trans-beta-ionone. ZL-13 and ZL-5 demonstrated similar aromatic profiles during development. Nonanal was found to unanimously provide the key beany flavor in the four varieties by serving as an important flavor contributor. Transcriptomic analysis revealed that ZL-13 exhibited the most pronounced time-dependent gene expression patterns with the highest number of up and downregulated DEGs among the four varieties. The coordinated downregulation of four *GGPS* genes during seed development was identified as a key factor influencing the linalool accumulation patterns in the studied varieties. Integration of the WGCNA results suggests that the accumulation of trans-beta-ocimene was likely regulated by the TFs from the RLK-Pelle, WRKY, AP2/ERF, bHLH, and bZIP families, while the MYB TFs may play crucial roles in modulating phenylpropanoid/benzenoid derivatives. The present work studied the VOC formation during seed development in four mung bean varieties, elucidated the molecular mechanisms underlying aroma profile formation, and established a scientific foundation for the development of improved mung bean-based ingredients.

## Figures and Tables

**Table 1:** The relative odor activity value (ROAV) of the VOCs.

Flavor Compounds	Flavor Threshold (*d*, mg·kg^−1^)	ROAV											
		LL655 BS	LL655 FS	LL655 BM	ZL-5 BS	ZL-5 FS	ZL-5 BM	ZL-13 BS	ZL-13 FS	ZL-13 BM	SH-1 BS	SH-1 FS	SH-1 BM
**Alcohols**													
1-Octen-3-ol	0.0015							7.81					
3-Methyl-1-butanol	0.004		46.91	26.59			48.47	19.25	18.67	66.43			
2-Methyl-1-butanol	0.0159		7.83						0.82				
2-Heptanol	0.065235		0.62										
cis-2-Penten-1-ol	0.98	<0.01			0.01	0.52	0.94	1.01					
**Aldehydes**													
Nonanal	0.0011	1.42	1.38	26.92	22.19	15.09		12.55	23.49	14.21	72.39	24.62	50.96
trans-2-Pentenal	0.98		0.01					0.05	0.01				
Methional	0.00045		33.93					14.56					
alpha-Tolualdehyde	0.0063		24.26	3.35				3.98	1.49				
**Ketone**													
Isopentyl methyl ketone	0.089									0.09			
Sulcatone	0.068				0.13			0.39					
**Alkanes**													
Nonane	4.8	<0.01											
**Terpenoids**													
*p*-Cymene	0.00501							1.94			4.35	2.98	
Linalool	0.00022	2.51	100.00								100.00	100.00	
Eucalyptol	0.0011	11.21		100.00	100.00	100.00	100.00	100.00	100.00	100.00	88.40	48.70	100.00
D-Limonene	0.034		1.58	0.42	0.54			0.21	0.44		11.72	3.23	0.85
beta-Pinene	0.14	<0.01											
Levomenthol	2.28		<0.01	<0.01							0.02		0.01
gamma-Terpinene	1							0.02					
(+)-2-Bornanone	1.36		0.01	0.01					<0.01	0.01	0.07	0.03	0.02
beta-Cyclocitral	0.05	0.02											
trans-beta-Ionone	0.000007	100.00											
alpha-Phellandrene	0.04	0.01			0.20			0.31					
trans-beta-Ocimene	0.034		0.14	0.23	0.60			0.47			0.67		0.40
**Phenylpropanoids/benzenoid derivatives**													
Toluene	0.527		0.40	1.00	1.44				0.68	1.67	1.36	1.24	2.06
o-Xylene	0.45023		0.09					0.04		0.05			
Ethylbenzene	2.20525		0.01	0.01			0.01	<0.01		0.02	0.04	0.02	0.02

Notes: BS: beginning seed; FS: full seed, BM: beginning maturity; and ROAV: relative odor activity value.

## Data Availability

The original contributions presented in the study are included in the article/Supplementary Material, further inquiries can be directed to the corresponding authors.

## Supplementary Materials

The following supporting information can be downloaded at: https://www.mdpi.com/article/10.3390/foods14132183/s1, Figure S1: The mung bean seed profiles at the three developmental stages. BS: beginning seeds; FS: full seeds; and BM: beginning maturity. Figure S2: Sample description of the transcriptomic analysis. A: Principal component analysis of the three developmental stages among the four mung bean varieties; B: correlation analysis of the biological duplicate samples. BS: beginning seeds; FS: full seeds; and BM: beginning maturity. Figure S3: The results of the expression genes in the four mung bean varieties. A: The number of expression genes at the three developmental stages; B: the classification of expression genes according to the FPKM in each mung bean variety; C: Venn plot of the expression genes; and D: Venn plot of the expression genes according to developmental stage in each mung bean variety. BS: beginning seeds; FS: full seeds; and BM: beginning maturity. Figure S4: The KEGG annotation results in each mung bean variety. I: cellular process; II: environmental information processing; III: genetic information processing; IV: metabolism; and V: organismal systems. Figure S5: The VOC profiles in each mung bean variety. BS: beginning seeds; FS: full seeds; and BM: beginning maturity. Figure S6: Annotation of key genes involving VOC formation. A: WGCNA results; B: correlation results between the modules and VOC; C: the KEGG classification in each module; and D: the number of genes in the relative pathways according to the module. Table S1: The classification of annotated TFs. Table S2: The flavor of the detected VOCs in the four mung bean varieties. Table S3: Correlation analysis between the MYB TFs and phenylpropanoid/benzenoid derivatives.

## Abbreviations

The following abbreviations are used in this manuscript:

VOC	Volatile organic compound
ROAV	Relative odor activity value
OAV	Odor activity value
TF	Transcription factor
BS	Beginning seed
FS	Full seed
BM	Beginning maturity
FW	Fresh weight
WGCNA	Weighted gene co-expression network analysis
PAC	Principal component analysis
DEG	Differentially expressed gene
TDC	L-tryptophan decarboxylase
PAO	Primary-amine oxidase
GGPP	Geranylgeranyl diphosphate
GGPS	Geranylgeranyl diphosphate synthase
GGAT	Glutamate-glyoxylate aminotransferase
CYP76F14	(E)-8-carboxylinalool synthase
LOX	Lipoxygenase
ALDH	Aldehyde dehydrogenase
