# Supplementary material for: Integrated Metabolomic and Transcriptomic Analysis of Volatile Organic Compound Biosynthesis During Mung Bean (Vigna radiata) Seed Development
Source: Foods. 2025 Jun 22;14(13):2183. doi: 10.3390/foods14132183 (PMC12248855; doi:10.3390/foods14132183)
Supplement: Supplementary file 1 [file foods-14-02183-s001.zip › Supplementary figure caption.pdf]

Figure S1. The mung bean seeds profiles at three developmental stages. BS: beginning seeds; FS: full seeds; BM: beginning maturity.

Figure S2. Sample description of transcriptomic analysis. A: Principal component analysis of three developmental stages among four mung bean varieties; B: Correlation results among biological duplicate samples. BS: beginning seeds; FS: full seeds; BM: beginning maturity.

Figure S3. The results of expression genes in four mung bean varieties. A: The number of expression genes at three developmental stages; B: The classification of expression genes according to FPKM at three developmental stages in each mung bean variety; C: Venn plot of expression genes; D: Venn plot of expression genes according to developmental stage in each mung bean variety. BS: beginning seeds; FS: full seeds; BM: beginning maturity.

Figure S4. KEGG annotation results in each mung bean variety. I: Cellular process; II: Environmental information processing; III: Genetic information processing; IV: Metabolism; V: Organismal systems.

Figure S5. VOC profiles in each mung bean variety. BS: beginning seeds; FS: full seeds; BM: beginning maturity.

Figure S6. Annotation of key genes involving VOC formation. A: WGCNA results; B: Correlation results between module and VOC; C: KEGG classification in each module. D: Number of genes in relative pathway according to module.
