# Supplementary material for: Integrated Metabolomic and Transcriptomic Analysis of Volatile Organic Compound Biosynthesis During Mung Bean (Vigna radiata) Seed Development
Source: Foods. 2025 Jun 22;14(13):2183. doi: 10.3390/foods14132183 (PMC12248855; doi:10.3390/foods14132183)
Supplement: Supplementary file 1 [file foods-14-02183-s001.zip › Table S2.pdf]

Table S2. The flavor of detected VOCs in four mung bean varieties.

Volatile Name	CAS No.	Category	Flavor Sources			Conclude Flavor
			PubChem	FEME	Perflavory	
1-Octen-3-ol	3391-86-4	alcohol	sweet, earthy	cucumber, earthy, fatty, floral, mushroomy	mushroomy, earthy, greeny, oily	earthy
3-Methyl-1-butanol	123-51-3	alcohol	alcohol	burnt, cocoa, floral, malt	oily, alcoholic, fruity	burnt
2-Methyl-1-butanol	137-32-6	alcohol	cooked roasted aroma with fruity or alcoholic undertones	fish oil, green, malt, onion, wine	fusel, alcoholic, fatty, greasy	burnt
2-Heptanol	543-49-7	alcohol	mild alcohol, lemon-like, grass-herbaceous	citrus, earth, fried, mushroom, oily	fruity, lemon, greeny, grassy, herbal	greeny
cis-2-Penten-1-ol	1576-95-0	alcohol			greeny, nasturtium	greeny
Nonanal	124-19-6	aldehyde	rose-orange	fatty, floral, greeny, lemon	waxy, citrus, cucumber, melon	citrus
trans-2-Pentenal	1576-87-0	aldehyde	pungent		pungent, greeny, fruity	pungent
Methional	3268-49-3	aldehyde	onion, meat-like	cooked potato, soy	musty, potato, tomato, earthy, vegetable	earthy
alpha-Tolualdehyde	122-78-1	aldehyde	green, floral, sweet	berry, geranium, honey, nut, pungent	greeny, sweet, floral hyacinth, clover, honey, cocoa	sweet
Isopentyl methyl ketone	110-12-3	ketone	fruity	-	-	fruity
Sulcatone	110-93-0	ketone	fatty, green, citrus	citrus, mushroomy, pepper, rubber, strawberry	citrus, greeny, musty lemongrass, apple	greeny
Nonane	111-84-2	alkane	gasoline-like	-	gasoline-like	gasoline-like
p-Cymene	99-87-6	terpenoid	citrus	citrus, fresh, solvent	citrus, woody, spicy	citrus

Linalool	78-70-6	terpenoid	floral	coriander, floral, lavender, lemon, rose	citrus, floral, sweet, woody	floral
Eucalyptol	470-82-6	terpenoid	camphor-like	camphor, cool, eucalyptol, mint	minty, herbal, camphor, medicinal	herbal
D-Limonene	5989-27-5	terpenoid	lemon-like	citrus, mint	citrus, orange, fresh, sweet	citrus
beta-Pinene	127-91-3	terpenoid	piney, turpentine-like	pine, polish, wood	dry, woody, pine	woody
Levomenthol	2216-51-5	terpenoid	peppermint	mint, cool	peppermint, cooling	herbal
gamma-Terpinene	99-85-4	terpenoid	lemon	bitter, citrus	oily woody, citrus, lemon, tropical fruity	citrus
(+)-2-Bornanone	464-49-3	terpenoid	camphor	camphor, earthy, pine, spicy	camphor, minty, herbal, woody	herbal
beta-Cyclocitral	432-25-7	terpenoid			tropical saffron herbal, tobacco, rose	herbal
trans-beta-Ionone	79-77-6	terpenoid	warm woody, dry	floral, violet	floral, woody, berry	woody
alpha-Phellandrene	99-83-2	terpenoid		citrus, minty, peppery, woody	citrus, herbal, greeny, woody, peppery	citrus
trans-beta-Ocimene	3779-61-1	terpenoid		floral	sweet, herbal	floral
Toluene	108-88-3	other	sweet, pungent		sweet	sweet
o-Xylene	95-47-6	other	sweet		geranium	sweet
Ethylbenzene	100-41-4	other	sweet, pungent			sweet
