# Supplementary figures and images for: Integrated Metabolomic and Transcriptomic Analysis of Volatile Organic Compound Biosynthesis During Mung Bean (Vigna radiata) Seed Development
Source: Foods. 2025 Jun 22;14(13):2183. doi: 10.3390/foods14132183 (PMC12248855; doi:10.3390/foods14132183)

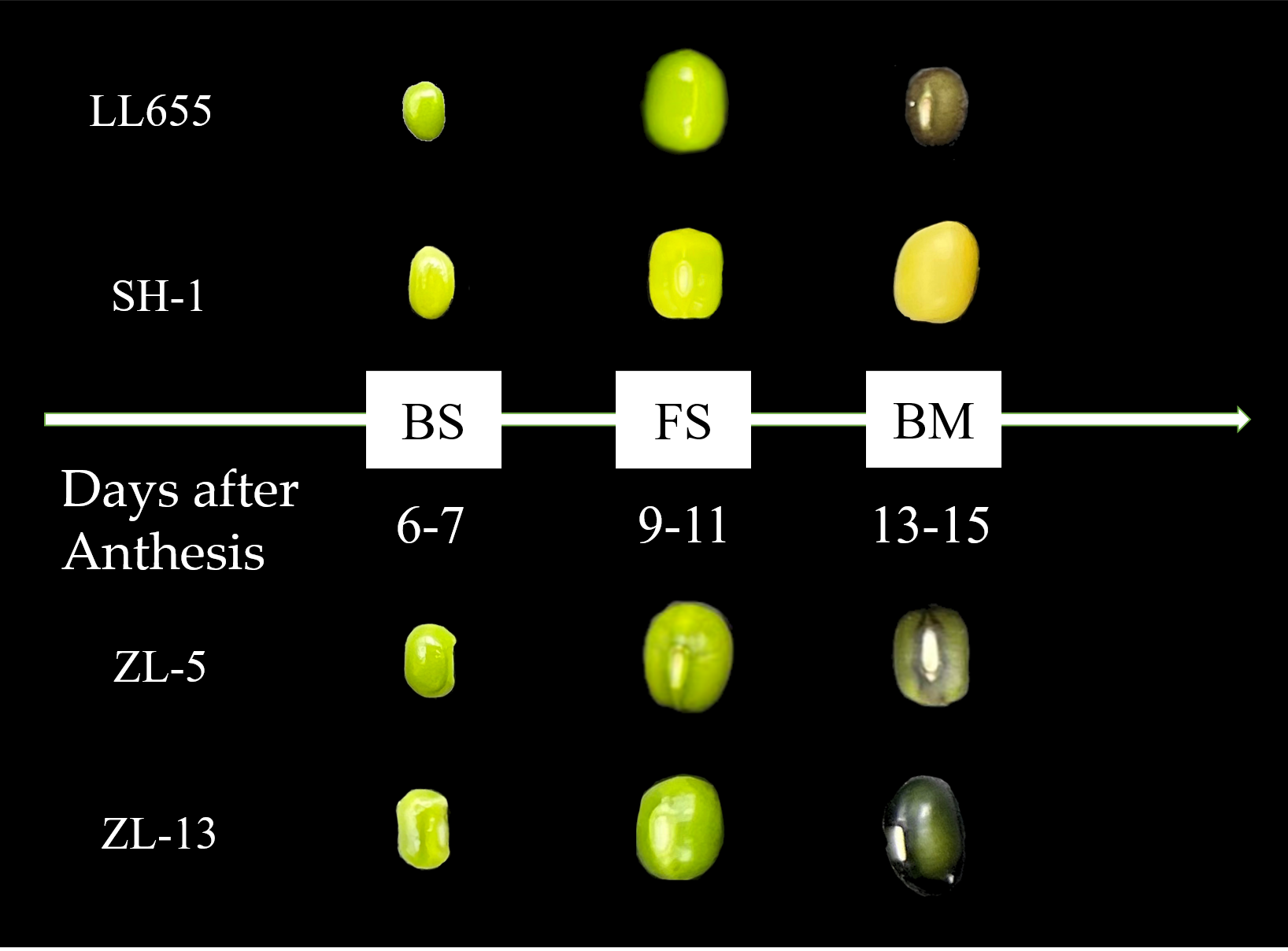

Supplement: Supplementary file 1 [file foods-14-02183-s001.zip › Figure S1.tif]

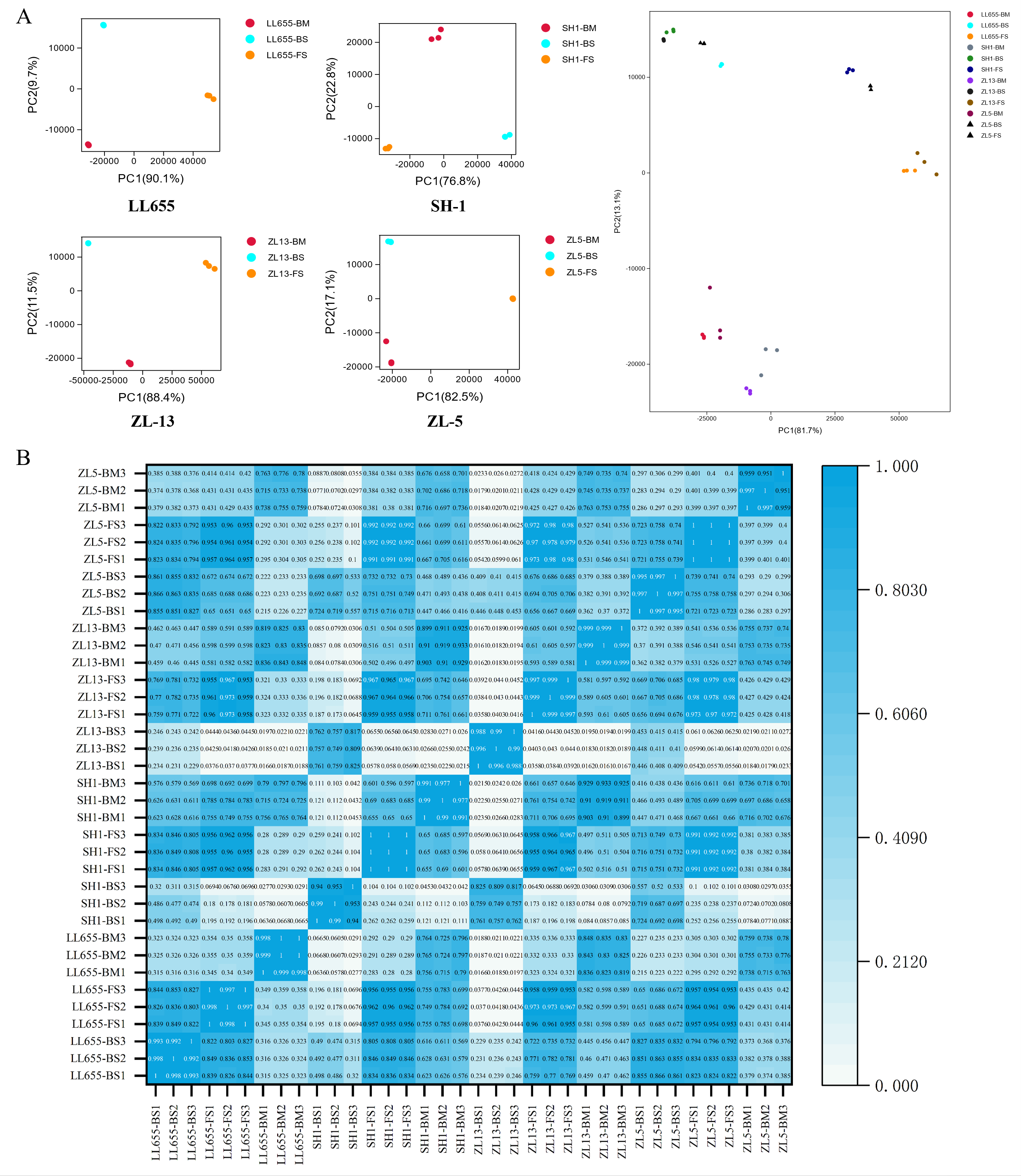

Supplement: Supplementary file 1 [file foods-14-02183-s001.zip › Figure S2.tif]

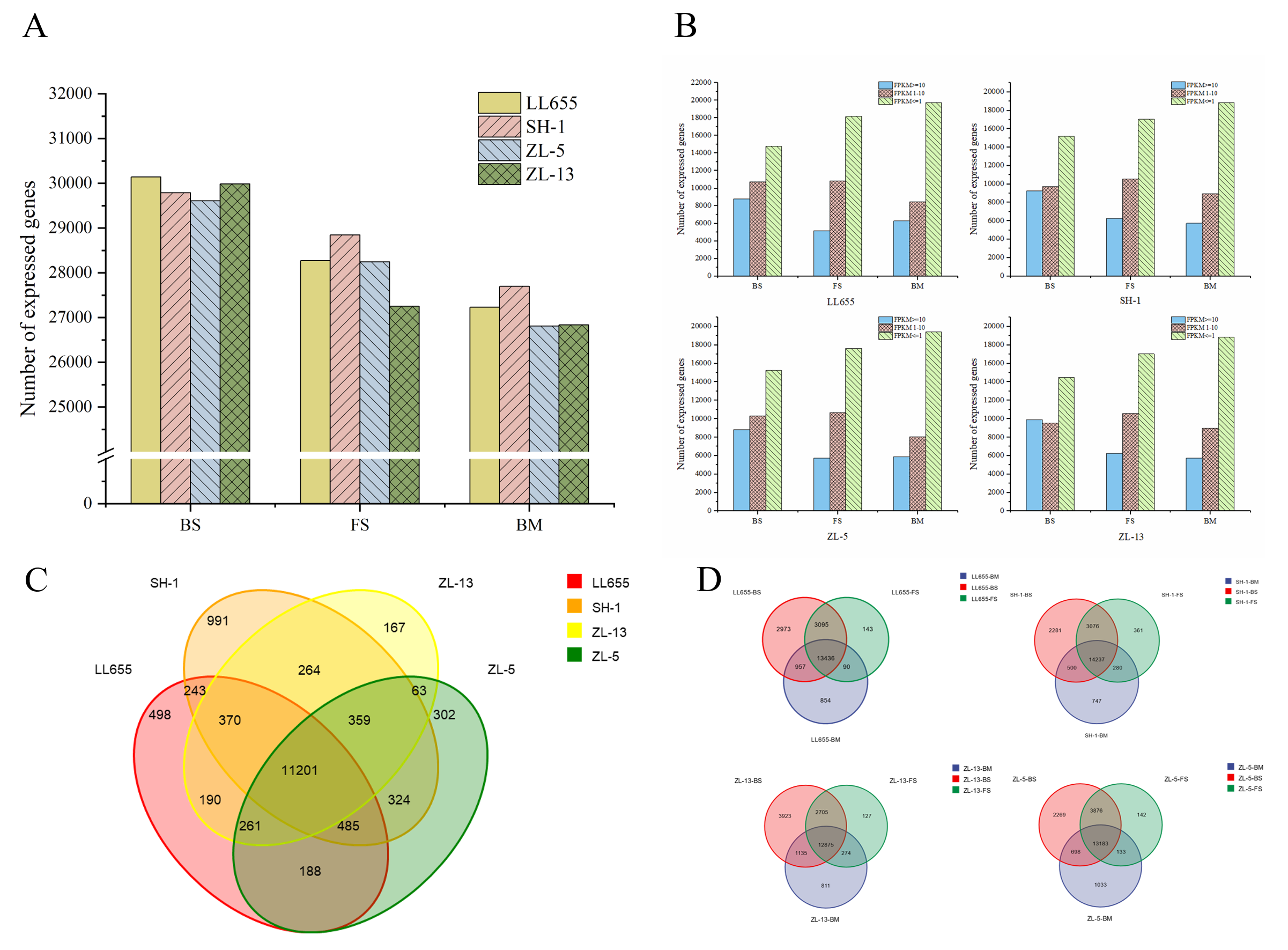

Supplement: Supplementary file 1 [file foods-14-02183-s001.zip › Figure S3.tif]

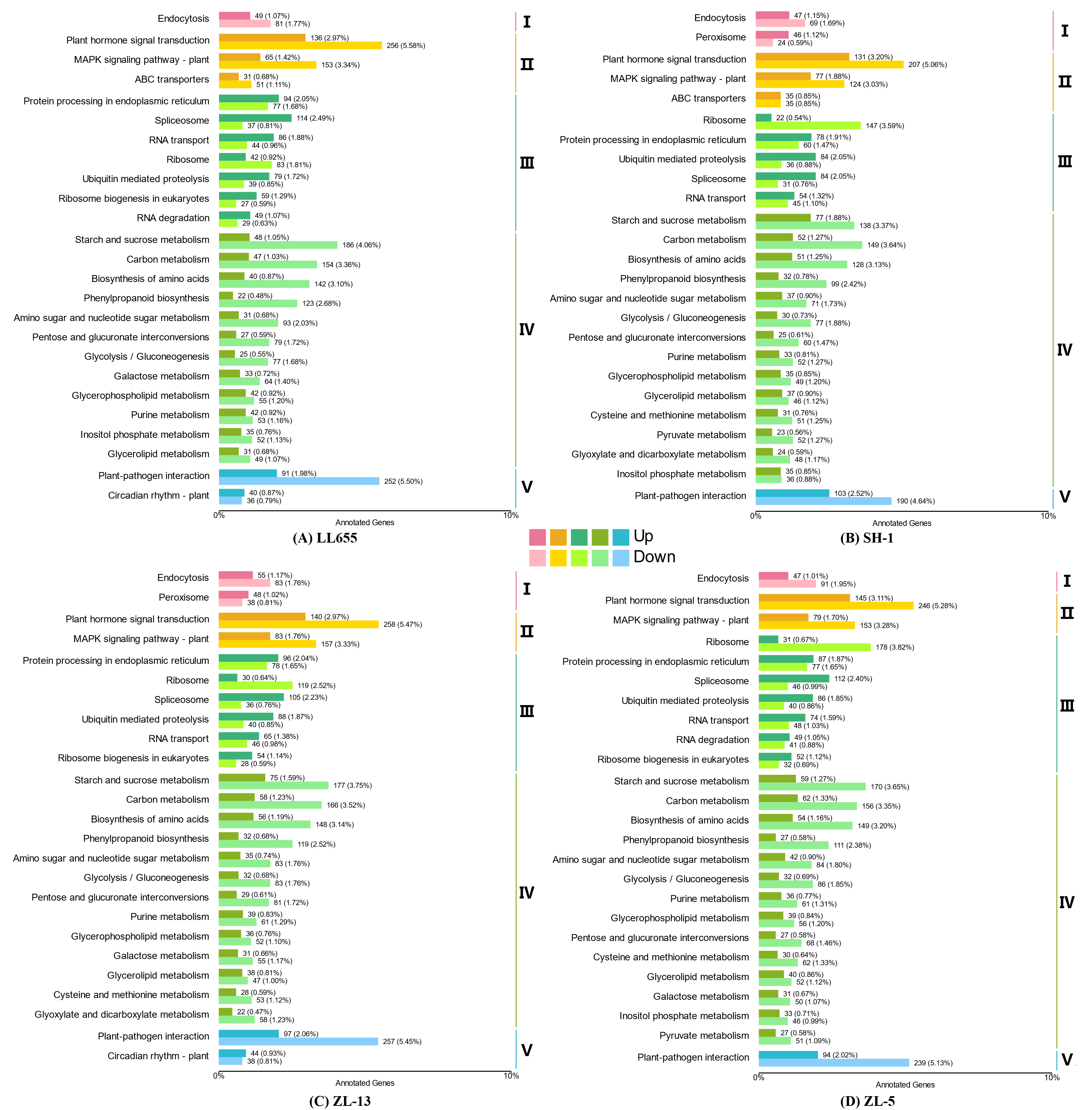

Supplement: Supplementary file 1 [file foods-14-02183-s001.zip › Figure S4.tif]

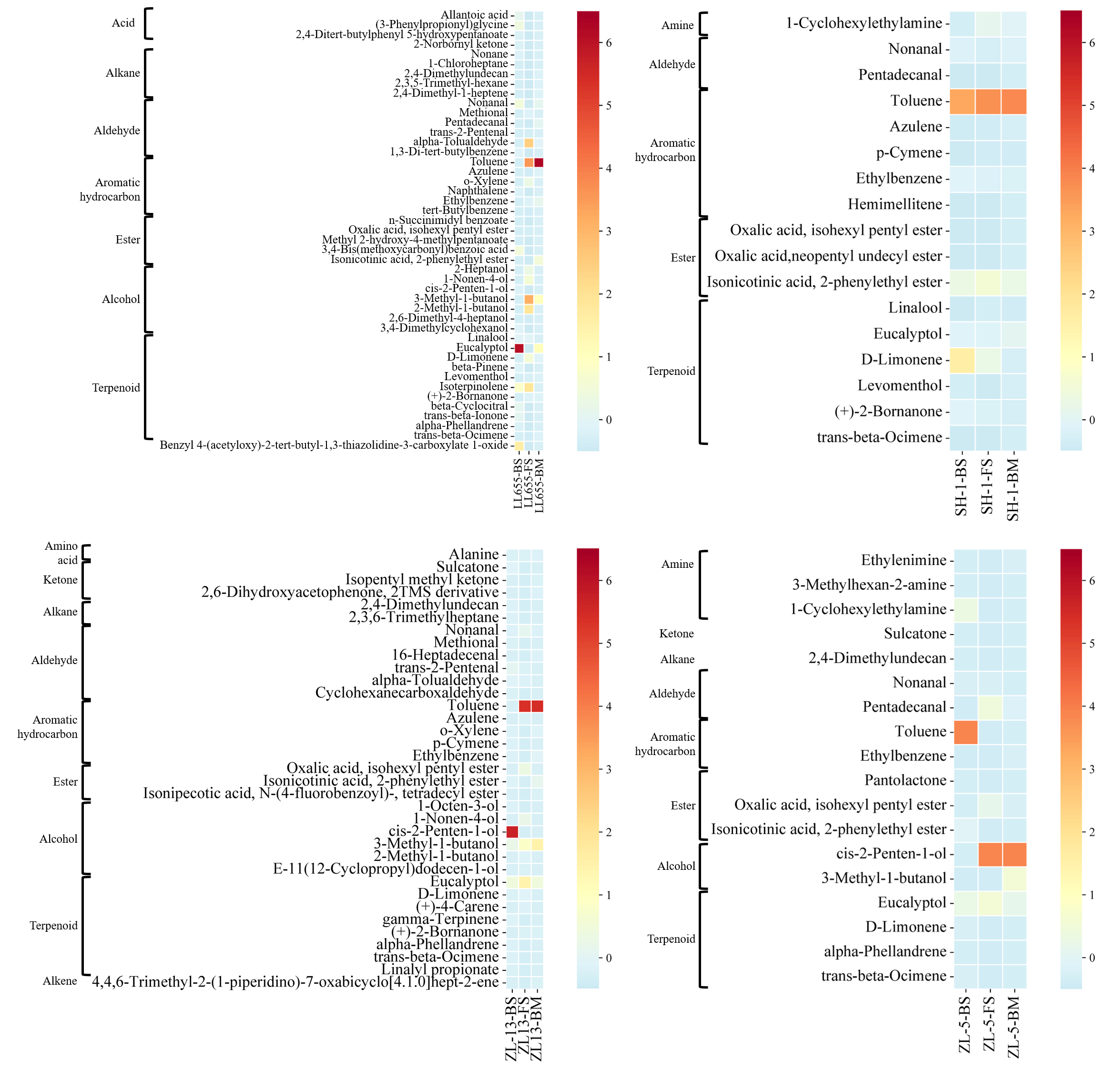

Supplement: Supplementary file 1 [file foods-14-02183-s001.zip › Figure S5.tif]

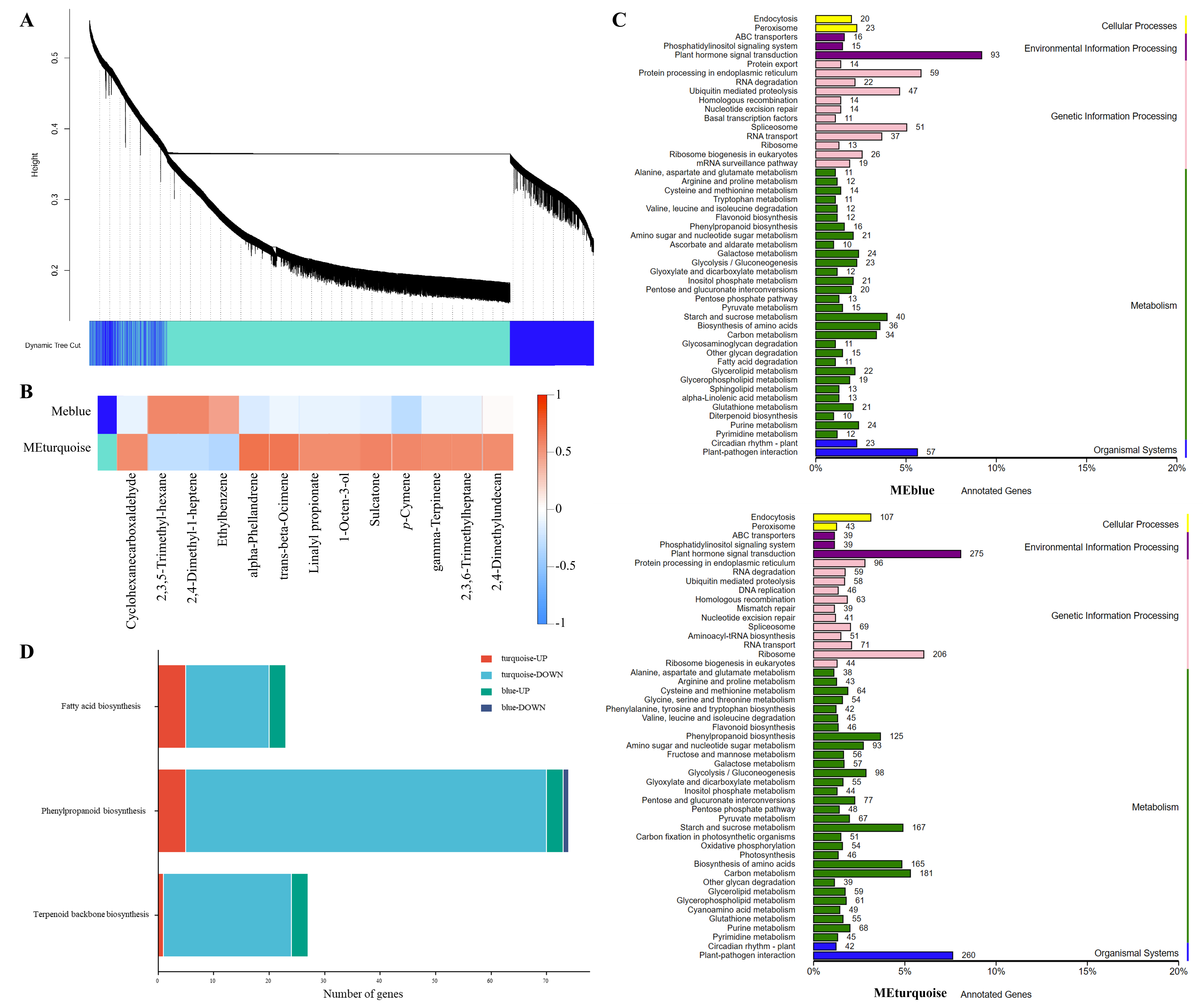

Supplement: Supplementary file 1 [file foods-14-02183-s001.zip › Figure S6.tif]
